# Topological Indices of Novel Drugs Used in Autoimmune Disease Vitiligo Treatment and Its QSPR Modeling

**DOI:** 10.1155/2022/6045066

**Published:** 2022-11-15

**Authors:** Saima Parveen, Nadeem Ul Hassan Awan, Fozia Bashir Farooq, Rakotondrajao Fanja, Qurat ul Ain Anjum

**Affiliations:** ^1^Department of Mathematics, Government College University Faisalabad, Pakistan; ^2^Department of Mathematics, Imam Muhammad Ibn Saud Islamic University, Riyadh, Saudi Arabia; ^3^Department of Mathematics and Informatics, Antananarivo University, BP 907 101 Antananarivo, Madagascar; ^4^Interdisciplinary Research Center in Biomedical Materials CIIT Lahore, Pakistan

## Abstract

A topological index is a real number derived from the structure of a chemical graph. It is helpful to determine the physicochemical and biological properties of a wide range of drugs, and it better reflects the theoretical properties of organic compounds. This is accomplished using degree-based topological indices. Vitiligo is a common, acquired skin pigmentation disorder that significantly impacts the quality of life. It frequently embodies a therapeutic challenge, resulting in interest in alternative treatments based on vitamin and herbal supplements. In this article, azathioprine, clobetasol, desonide, hydrocortisone valerate, and other drugs utilized to cure vitiligo have discoursed, and the goal of QSPR revision is to determine the mathematical relationship between properties under investigation (e.g., polarity and enthalpy) and diverse descriptors associated with the drugs' molecule. The QSPR model will help to predict physical properties. In this study, topological indices (TIs) imposed on said drugs were found to have a good correlation with physicochemical properties in this course. Finally, this work can be helpful to design and synthesize new vitiligo treatments and other disease drugs.

## 1. Introduction

**Figure 1:**
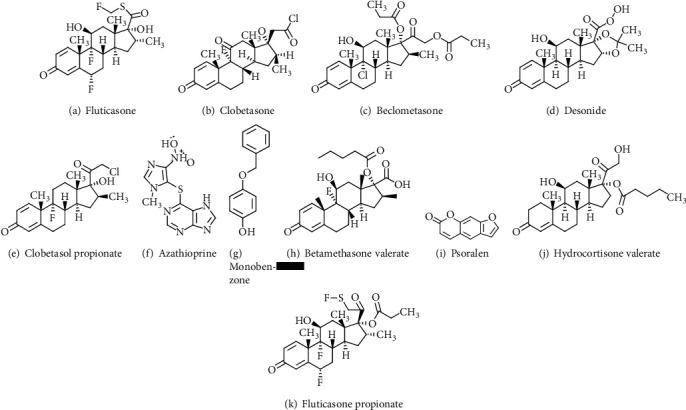
Molecular structure of drugs.

Vitiligo is a familiar depigmenting skin disorder characterized by idiopathic, acquired, gradual, delimited hypomelanosis of the hair and skin, with a total absence of melanocytes under the microscope. Vitiligo is a serious skin disease that affects the patient's quality of life significantly. [1]. The disease is characterized by melanocyte loss and the development of depigmented patches, which results in pigment dilution in the affected skin areas. It occurs globally, with incidence rates ranging from 0.5 to 4%, and its prevalence is comparable across genders and races [2]. Significant progress in understanding the pathogenesis of vitiligo has been made, and today, it is certainly categorized as an autoimmune disease [3]. Vitiligo ought not to be ignored as a minor or insubstantial disease, as its consequences can be psychologically catastrophic, causing profound emotional distress and, in many cases, a significantly reduced quality of daily life. Vitiligo patients may feel self-conscious or anxious about their skin. They can be rude at times, such as staring or saying hurtful things. This, in turn, may cause anxiety. Patients are most vulnerable to the disease's negative psychosocial impact when they are between the ages of 10 and 30. It is quite often a therapeutic challenge, prompting attention in therapeutic options such as herbal and vitamin supplements. Medicos and scientists are constantly searching for more effective methods to treat vitiligo patients. One approach is to develop and test new drugs. Drug discovery is a hard process because it is expensive, time consuming, and in certain cases extremely difficult. Drugs are prescribed to treat and halt the said fatal disease, and numerous drug tests are conducted to combat the fatal disease. This necessitates prompt medical assessment, screening, and medication to assist patients in disease control. The eleven vital drugs, medicines like fluticasone propionate, clobetasone, beclomethasone dipropionate, desonide, azathioprine, clobetasol propionate, monobenzone, fluticasone, betamethasone valerate, psoralen, and hydrocortisone valerate, are safe and effective medicines that are compelled to ensure the health of the community. The chemical structure of the aforementioned drugs is depicted in Figure 1.

Topological indices (TIs) are quantitative descriptors derived from a chemical graph that completely describes the chemical system and is extensively used in the research project on several drugs' physicochemical properties. Because polynomials and TIs are widely assessed and represent the chemical structure, they play an important role in chemical graph theory. Degree-based TIs are crucially significant and play a key role in chemical graph theory. There has been a lot of interest in the use of graph invariants (TIs) in quantitative structure-property relationships (QSPR) and quantitative structure-activity relationships (QSAR) studies over the last few years. TIs have applications in numerous areas of mathematics, such as bioinformatics, mathematics, informatics, and biology, but their most useful aspect to date has taken place in nonempirical QSPR [4]. Drug bioactivity can be predicted using the ABC index, Wiener index, and Randic index. The QSPR models aid in determining the most appropriate relationship between topological indices and psychochemical properties. These psychochemical properties are being examined because they all have a major impact on bioactivity and drug transport in the human body. We calculated degree-based TIs for vitiligo drugs in this paper. Likewise, vitiligo drugs are organic molecules with carefully defined topological indices that undergo purposeful QSPR analysis. The respective characteristic approximated by this method is highly correlated with the characteristic of vitiligo drugs using linear regression. There is a strong correlation between drug properties and TIs, which has been discovered.

Previous research on potential drugs against COVID-19 is discussed by Colakoglu [5]. Novel drugs used in cancer treatment were discussed by Havare [6] and discussed that drug discovery is a costly and complex phenomenon, so these are best predicted with this method. Blood cancer drug QSPR modeling [3] shows a strong correlation between TIs and drug properties. Advances in QSPR studies for various topological indices for various chemical structures motivated us to work on the current research problem. The purpose of this study is to look into the use of TIs in determining the physical properties and its QSPR modeling of vitiligo disease drug regimens used in therapeutic management.

Previous research on potential drugs against COVID-19 is discussed by Colakoglu [7]. Novel drugs used in cancer treatment were discussed by Havare [6] and discussed that drug discovery is a costly and complex phenomenon, so these are best predicted with this method. Blood cancer drug QSPR modeling was done by Nasir et al. [8] which shows a strong correlation between TIs and drug properties. Advances in QSPR studies for various topological indices for various chemical structures motivated us to work on the current research problem. The purpose of this study is to look into the use of TIs in determining the physical properties and its QSPR modeling of vitiligo disease drug regimens used in therapeutic management. Rheumatoid arthritis (RA) is a joint disease, according to Parveen et al. [9]. They used purposeful QSPR analysis and carefully crafted topological indexes to investigate the chemical components that make up RA medications. A computer method was put out by Sakander et al. [10] for computing analytically precise equations for specific degree and distance-based topological indices for generic networks. In order to demonstrate that our technique is more effective and has less algorithmic and computational complexity, some experiments are carried out in comparison to the well-known techniques. Four polynomials, Sadhana, omega, theta, and Padmakar–Ivan for double benzenoid chains, are calculated by Fozia et al. [11]. These polynomials' analytical closed expressions are derived using the edge-cut approach. The QSPR modeling of antituberculosis drugs is detailed in [12], and Parveen et al. [13] completed the QSPR study of diabetes treatments and found a best fit model for it.

## 2. Material and Method

In drug configuration, atoms depict vertices, and the associated bonds connecting the atoms are termed to as edges. Graph *G*(*V*, *E*) is thought to be simple, finite, and connected, whereas *V* and *E* in the chemical graph are referred to as vertex and the edge set, respectively. The degree of a graph vertex is the number of vertices adjacent to *G* is denoted by *d*_*u*_. In chemistry, the valence of a compound and the degree of a vertex in a graph are concepts that are inextricably linked [4, 14–16]. We used the following degree-based topological indices:

**Definition 1 .**

The ABC index [17] of *G* is defined as follows:

(1) $$\begin{array}{c} ABC\left(G\right)=\underset{uv\in E\left(G\right)}{\sum }\sqrt{\frac{d_{u+}d_{v}-2}{d_{u}d_{v}}}. \end{array}$$

**Definition 2 .**

The first TI is Randic index *RA*(*G*) introduced by Milan Randic [18]. Randic index is defined as follows:

(2) $$\begin{array}{c} RA\left(G\right)=\underset{uv\in E\left(G\right)}{\sum }\sqrt{\frac{1}{d_{u}d_{v}}}. \end{array}$$

**Definition 3 .**

The sum connectivity index [15] of *G* is defined as follows:

(3) $$\begin{array}{c} S\left(G\right)=\underset{uv\in E\left(G\right)}{\sum }\sqrt{\frac{1}{d_{u}+d_{v}}}. \end{array}$$

**Definition 4 .**

The GA index [19] of *G* is defined as follows:

(4) $$\begin{array}{c} GA\left(G\right)=\underset{uv\in E\left(G\right)}{\sum }\frac{2\sqrt{d_{u}d_{v}}}{d_{u}+d_{v}}. \end{array}$$

**Definition 5 .**

The first and second Zagreb indices [20] of *G* is defined as follows:

(5) $$\begin{array}{c} M_{1}\left(G\right)=\underset{uv\in E\left(G\right)}{\sum }\left(d_{u}+d_{v}\right),\\ M_{2}\left(G\right)=\underset{uv\in E\left(G\right)}{\sum }\left(d_{u}d_{v}\right). \end{array}$$

**Definition 6 .**

The harmonic index [21] of *G* is defined as follows:

(6) $$\begin{array}{c} H\left(G\right)=\underset{uv\in E\left(G\right)}{\sum }\frac{2}{d_{u}+d_{v}}. \end{array}$$

**Definition 7 .**

The hyper Zagreb index [22] of *G* is defined as follows:

(7) $$\begin{array}{c} HM\left(G\right)=\underset{uv\in E\left(G\right)}{\sum }{\left(d_{u}+d_{v}\right)}^{2}. \end{array}$$

**Definition 8 .**

The forgotten index [23] of *G* is defined as follows:

(8) $$\begin{array}{c} F\left(G\right)=\underset{uv\in E\left(G\right)}{\sum }\left[{\left(d_{u}\right)}^{2}+{\left(d_{v}\right)}^{2}\right]. \end{array}$$

The *π*-electron energy of a molecule was calculated using the first and second Zagreb indices [16]. The heat of the creation of alkanes can be preeminently predicted using the augmented Zagreb index [24]. ChemSpider is used to calculate the values of physical properties.

Table 1 shows that the data is normally distributed. As a result, the linear regression model is best to check and use for the aforementioned analysis. We endorse that readers read the following articles [3, 6, 14, 24–26]. Monobenzone propionate has a molecular formula of C_13_H_12_O_2_. It is a hydroquinone derivative that is used in the treatment of vitiligo. It is the monobenzone ether of hydroquinone, which is used in medicine to treat pigmentation. This medication comes in the form of a white, nearly tasteless white crystalline that is soluble in organic solvents but practically insoluble in water. It has a depigmenting effect on mammalian skin by increasing melanin excretion from melanocytes. It may also cause melanocyte destruction and permanent depigmentation. Monobenzone works by effectively removing colour from normal skin around vitiligo skin. Fluticasone has the molecular formula of C_22_H_27_F_3_O_4_S. It cures corticosteroid-responsive dermatoses. Clobetasone has the formula of C_22_H_26_ClFO_4_. It is frequently used topically as a treatment for a variety of ailments. It is often employed topically as a treatment for a variety of conditions such as eczema, various forms of dermatitis, psoriasis, and for certain ophthalmologic conditions. When cortisol derivatives are applied to the skin, they produce anti-inflammatory, antiproliferative, immunosuppressive, and vasoconstrictor effects. Topical clobetasone butyrate is used in dermatology to heal itchiness and erythema caused by eczema and dermatitis. Clobetasone and its metabolites are eliminated through the urine. Beclomethasone dipropionate has the molecular formula of C_28_H_37_N_7_ClO_7_. In 1972, it was first available in a pressurized metered-dose inhaler, followed by a dry powder inhaler and an aqueous nasal spray. Beclomethasone dipropionate is used to treat inflammatory conditions such as asthma, dermatoses, and allergic rhinitis because of its anti-inflammatory, antipruritic, and antiallergy properties and excreted in urine. Desonide has a molecular formula of C_24_H_32_O_6_. It is a nonfluorinated synthetic corticosteroid used topically in dermatology.

Corticosteroids are a group of steroids and used as anti-inflammatory and antipruritic agents. Betamethasone is used to relieve inflammation in several conditions such as an allergic and dermatologic disorder. It topically manages inflammatory skin conditions including autoimmune disorder. Clobetasol propionate has the molecular formula of C_25_H_32_ClFO_5_. It is a corticosteroid that is used to treat corticosteroid-responsive dermatomes as well as plaque psoriasis. Azathioprine propionate has the molecular formula of C_9_H_7_N_7_O_2_S. It is an immunosuppressant that is helpful to reduce Crohn's disease, rheumatoid arthritis, and ulcerative colitis and also to prevent renal transplant rejection. It is used to treat inflammatory diseases such as rheumatoid arthritis. Hydrocortisone valerate has the molecular formula of C_26_H_38_O_6_. It is a corticosteroid that is used to treat pruritic dermatoses and inflammation that are responsive to corticosteroids. It is also employed in the treatment of endocrine (hormonal) disorders. It is also used to treat a variety of allergic and immune conditions, including severe asthma, severe psoriasis, arthritis, and lupus. Psoralen is the parent chemical substance in a group of organic compounds in nature that are employed to heal vitiligo. Fluticasone propionate has the molecular formula of C_25_H_31_F_3_O_5_S. This is a glucocorticoid medication that is used to treat asthma, inflammatory pruritic dermatoses, and nonallergic rhinitis.

## 3. Results and Discussions

In this section, degree-based TIs are executed on vitiligo drugs. The relation between QSPR analysis and topological indices portrays that the properties are vastly correlated in terms of physicochemical properties for the said disease. The eleven medicines, fluticasone propionate, clobetasone, beclomethasone dipropionate, desonide, azathioprine, clobetasol propionate, monobenzone, fluticasone, betamethasone valerate, psoralen, and hydrocortisone valerate, are used in the analysis for vitiligo. The drug structures are displayed in Figure 1. We consider the molecular structure as graph, and the drug elements denote vertices and bonds among atoms are their edges. We use regression analysis calculation for drug study.

### 3.1. Regression Model

In this article, drug computable structure analysis of nine topological indices for QSPR modeling tenacity is performed. The five physical properties, refractivity (R), polarity, complexity, molar volume (MV), and enthalpy (E) for eleven medicines used in vitiligo treatment, are listed in Table 2. We conduct the regression analysis for the drugs, and the linear regression model is tested using the following equation:

(9) $$\begin{array}{c} P=A+b\;\left(TI\right), \end{array}$$

where *P* denotes the physicochemical property of the given drug. The *TI* stands for topological index, *A* stands for constant, and *b* stands for regression coefficient. The Statistix, SageMath, and MATLAB software are useful for determining the results. A linear QSPR model is used to analyze nine TIs of vitiligo drugs and their physiochemical properties. Equation (9) is pertinent for the aforementioned calculation.

**Theorem 1 .**

Let *G*_1_ be the graph Psoralen. The various TIs of *G*_1_ are given as follows:

(10) $$\begin{array}{c} ABC\left(G_{1}\right)=11.34,\\ RA\left(G_{1}\right)=6.82,\\ S\left(G_{1}\right)=7.29,\\ GA\left(G_{1}\right)=15.66,\\ M_{1}\left(G_{1}\right)=78,\\ M_{2}\left(G_{1}\right)=93,\\ F\left(G_{1}\right)=200,\\ HM\left(G_{1}\right)=386,\\ H\left(G_{1}\right)=6.67. \end{array}$$

**Proof**

Let *G*_1_ be graph of psoralen and let *E*_*m*,*n*_ represent the class of edges of *G*_1_  joining vertices with |*E*_1,3_| = 1, |*E*_2,2_| = 3, |*E*_2,3_ | = 10, and |*E*_3,3_| = 2.

- By using Definition 1, we get the following:

(11) $$\begin{array}{c} ABC\left(G_{1}\right)=\sqrt{\frac{1+3-2}{1\times 3}\;}+3\sqrt{\frac{2+2-2}{2\times 2}\;}+10\sqrt{\frac{2+3-2}{2\times 3}\;}+2\sqrt{\frac{3+3-2}{3\times 3}\;}=11.34. \end{array}$$

(ii) By using Definition 2, we get the following:

(12) $$\begin{array}{c} RA\left(GG_{1}\right)=\sqrt{\frac{1}{1\times 3}\;}++3\sqrt{\frac{1}{2\times 2}\;}+10\sqrt{\frac{1}{2\times 3}\;}+2\sqrt{\frac{1}{3\times 3}\;}=6.82. \end{array}$$

(iii) By using Definition 3, we get the following:

(13) $$\begin{array}{c} S\left(G_{1}\right)=\sqrt{\frac{1}{1+3}\;}+3\sqrt{\frac{1}{2+2}\;}+10\sqrt{\frac{1}{2+3}\;}+2\sqrt{\frac{1}{3+3}\;}=7.29. \end{array}$$

(iv) By using Definition 4, we get the following:

(14) $$\begin{array}{c} GA\left(G_{1}\right)=\frac{\sqrt{1\times 3}}{1+3}+\frac{3\sqrt{2\times 2}}{2+2}+\frac{10\sqrt{2\times 3}}{2+3}+\frac{2\sqrt{3\times 3}}{3+3}=15.66. \end{array}$$

(v) By using Definition 5, we get the following:

(15) $$\begin{array}{c} M_{1}\left(G_{1}\right)=\left(1+3\right)+3\left(2+2\right)+10\left(2+3\right)+2\left(3+3\right)=78. \end{array}$$

(vi) By using Definition 5 and above given edge partitions *E*_*m*,*n*_, we get the following:

(16) $$\begin{array}{c} M_{2}\left(G_{1}\right)=\left(1\times 3\right)+3\left(2\times 3\right)+10\left(2\times 3\right)+2\left(3\times 3\right)=93. \end{array}$$

(vii) By using Definition 6, we get the following:

(17) $$\begin{array}{c} H\left(G_{1}\right)=\left(\frac{1}{1+3}\right)+3\left(\frac{1}{2+2}\right)+10\left(\frac{1}{2+3}\right)+2\left(\;\frac{1}{3+3}\right)=6.67. \end{array}$$

(viii) By using Definition 7, we get the following:

(18) $$\begin{array}{c} HM\left(G_{1}\right)={\left(1+3\right)}^{2}+3{\left(2+2\right)}^{2}+10{\left(2+3\right)}^{2}+2{\left(3+3\right)}^{2}=336. \end{array}$$

(ix) By using Definition 8, we get the following:

(19) $$\begin{array}{c} F\left(G_{1}\right)=\left(1+9\right)+3\left(4+4\right)+10\left(4+9\right)+2\left(9+9\right)=200. \end{array}$$

**Theorem 2 .**

Let *G*_2_ be graph of azathioprine. The various topological indices of *G*_2_ are given as follows:

(20) $$\begin{array}{c} ABC\left(G_{2}\right)=14.08,\\ RA\left(G_{2}\right)=8.79,\\ S\left(G_{2}\right)=9.24,\\ GA\left(G_{2}\right)=19.68,\\ M_{1}\left(G_{2}\right)=96,\\ M_{2}\left(G_{2}\right)=115,\\ F\left(G_{2}\right)=244,\\ H\left(G_{2}\right)=8.60,\\ HM\left(G_{2}\right)=474. \end{array}$$

**Proof**

Let *G*_2_ be the graph of azathioprine and let *E*^*ˊ*^_(*m*, *n*)_ represent the class of edges of  *G*_2_ joining vertices with |*E*_1,2_ | = 1,  |*E*_1,3_ | = 1, |*E*_2,2_ | = 5, |*E*_2,3_ | = 9, and |*E*_3,3_ | = 4.

- By using Definition 1, we get the following:

(21) $$\begin{array}{c} ABC\left(G_{2}\right)=\sqrt{\frac{1+2-2}{1\times 2}\;}+\sqrt{\frac{1+3-2}{1\times 3}\;}+5\sqrt{\frac{2+2-2}{2\times 2}\;}+9\sqrt{\frac{2+3-2}{2\times 3}\;}+4\sqrt{\frac{3+3-2}{3\times 3}\;}=14.08. \end{array}$$

(ii) By using Definition 2, we get the following:

(22) $$\begin{array}{c} RA\left(G_{2}\right)=\sqrt{\frac{1}{1\times 2}\;}+\sqrt{\frac{1}{1\times 3}\;}+5\sqrt{\frac{1}{2\times 2}\;}+9\sqrt{\frac{1}{2\times 3}\;}+4\sqrt{\frac{1}{3\times 3}\;}=8.79. \end{array}$$

(iii) By using Definition 3, we get the following:

(23) $$\begin{array}{c} S\left(G_{2}\right)=\sqrt{\frac{1}{1+2}\;}+\sqrt{\frac{1}{1+3}\;}+5\sqrt{\frac{1}{2+2}\;}+9\sqrt{\frac{1}{2+3}\;}+4\sqrt{\frac{1}{3+3}\;}=9.24. \end{array}$$

(iv) By using Definition 4, we get the following:

(24) $$\begin{array}{c} GA\left(G_{2}\right)=\frac{\sqrt{1\times 2}}{1+2}+\frac{\sqrt{1\times 3}}{1+3}+\frac{5\sqrt{2\times 2}}{2+2}+\frac{9\sqrt{2\times 3}}{2+3}+\frac{4\sqrt{3\times 3}}{3+3}=19.68. \end{array}$$

(v) By using Definition 5, we get the following:

(25) $$\begin{array}{c} M_{1}\left(G_{2}\right)=\left(1+2\right)+\left(1+3\right)+5\left(2+2\right)+9\left(2+3\right)+4\left(2+3\right)=96. \end{array}$$

(vi) By using Definition 5, we get the following:

(26) $$\begin{array}{c} M_{2}\left(G_{2}\right)=\left(1\times 2\right)+\left(1\times 3\right)+5\left(2\times 2\right)+9\left(2\times 3\right)+4\left(3\times 3\right)=115. \end{array}$$

(vii) By using Definition 6, we get the following:

(27) $$\begin{array}{c} H\left(G_{2}\right)=\left(\frac{1}{1+2}\right)+\left(\frac{1}{1+3}\right)+5\left(\frac{1}{2+2}\right)+9\left(\frac{1}{2+3}\right)+4\left(\frac{1}{3+3}\right)=9.60. \end{array}$$

(viii) By using Definition 7, we get the following:

(28) $$\begin{array}{c} HM\left(G\right)={\left(1+2\right)}^{2}+{\left(1+3\right)}^{2}+5{\left(2+2\right)}^{2}+9{\left(2+3\right)}^{2}+4{\left(3+3\right)}^{2}=474. \end{array}$$

(ix) By using Definition 8, we get the following:

(29) $$\begin{array}{c} F\left(G_{2}\right)=\left(1+4\right)+\left(1+9\right)+5\left(4+4\right)+9\left(4+9\right)+4\left(9+9\right)=244. \end{array}$$

**Figure 2:**
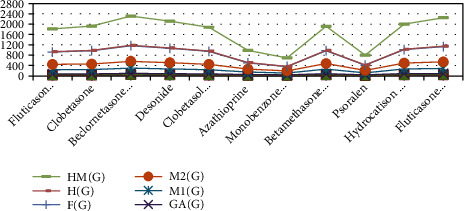
Medicines with TIs.

Topological indices for the remaining drugs can be calculated using the same procedure as in Theorems 9 and 10 and Definitions 1–8. To reduce the length of paper, only two drug calculations are added.  Table 1 also includes the calculated values for all drugs' TIs.  Figure 2 depicts a graphical representation of calculated TIs for various medicines. Using Equation (9), we calculated the getting-ready linear models for all TIs, which are listed as follows:

1. Regression models for *ABC* (*G*):

Enthalpy =46.897 + 2.171 [*ABC* (*G*)]

Polarity=6.498 + 1.426 [*ABC* (*G*)]

Molar volume=2.954 + 13.765 [*ABC* (*G*)]

Complexity=−309.258 + 50.191 [*ABC* (*G*)]

Refractivity =14.804 + 4.199 [*ABC* (*G*)]

(2) Regression models for *RA* (*G*):

Enthalpy=−2.826 + 6.871 [*RA* (*G*)]

Polarity=5.422 + 2.676 [*RA* (*G*)]

Molar volume=−1.826 + 23.293 [*RA* (*G*)]

Complexity=−321.914 + 84.555 [*RA* (*G*)]

Refractivity=11.323 + 7.267 [*RA* (*G*)]

(3) Regression models for *S* (*G*):

Enthalpy=−4.082 + 6.671 [*S* (*G*)]

Polarity=4.913 + 2.599 [*S* (*G*)]

Molar volume=−5.918 + 22.061 [*S* (*G*)]

Complexity=−338.894 + 82.205 [*S* (*G*)]

Refractivity=10.675 + 7.010 [*S* (*G*)]

(4) Regression models for *GA* (*G*):

Enthalpy=−3.676 + 3.112 [*GA* (*G*)]

Polarity=5.068 + 1.213 [*GA* (*G*)]

Molar volume=−4.316 + 10.537 [*GA* (*G*)]

Complexity=−335.797 + 38.423 [*GA* (*G*)]

Refractivity=11.940 + 3.243 [*GA* (*G*)]

(5) Regression models for *M*_1_(*G*):

Enthalpy =5.191 + 0.514 [*M*_1_(*G*)]

Polarity=9.228 + 0.196 [*M*_1_(*G*)]

Molar volume=30.594 + 1.708 [*M*_1_(*G*)]

Complexity=−214.531 + 6.269 [*M*_1_(*G*)]

Refractivity=24.456 + 0.513 [*M*_1_(*G*)]

(6) Regression models for *HM* (*G*):

Enthalpy =13.232 + 0.085 [*HM* (*G*)]

Polarity=12.603 + 0.032 [*HM* (*G*)]

Molar volume=59.517 + 0.279 [*HM* (*G*)]

Complexity=−111.88 + 1.029 [*HM* (*G*)]

Refractivity=33.988 + 0.083 [*HM* (*G*)]

(7) Regression models for *M*_2_(*G*):

Enthalpy =11.592 + 0.374 [*M*_2_(*G*)]

Polarity=11.709 + 0.142 [*M*_2_(*G*)]

Molar volume=52.287 + 1.240 [*M*_2_(*G*)]

Complexity=−138.898 + 4.569 [*M*_2_(*G*)]

Refractivity=31.763 + 0.368 [*M*_2_(*G*)]

(8) Regression models for *F* (*G*):

Enthalpy =57.317 + 0.079 [*F*(*G*)]

Polarity=14.324 + 0.50 [*F*(*G*)]

Molar volume=65.7780 + 0.058 [*F*(*G*)]

Complexity=−88.525 + 1.870 [*F*(*G*)]

Refractivity=35.904 + 0.150 [*F*(*G*)]

(9) Regression models for *H* (*G*):

Enthalpy =43.150 + 4.138 [*H*(*G*)]

Polarity=1.974 + 2.891 [*H*(*G*)]

Molar volume=−11.641 + 25.466 [*H*(*G*)]

Complexity=−355.677 + 92.284 [*H*(*G*)]

Refractivity=7.904 + 7.986 [*H* (*G*)]

Tables 3–11 represent the statistical parameters used in QSPR models of TIs.

### 3.2. Quantitative Structure Analysis and Comparison between Topological Indices and Correlation Coefficient of Physicochemical Properties

**Figure 3:**
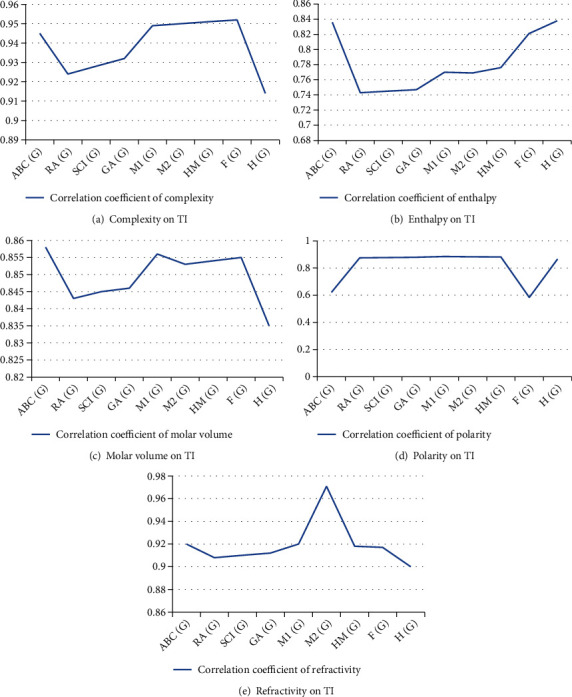
Physicochemical properties and TIs.

Table 2 shows physical properties of eleven vitiligo drugs, and Table 1 shows TIs computed using molecular structure.  Table 12 lists correlation coefficients between five physical properties and TIs.  Figure 3 depicts the graph between TIs and physical properties.

### 3.3. Calculation of Statistical Parameters

In this section, we find the relation between degree-based TIs and physical properties of vitilgo drugs such as medicines fluticasone propionate, clobetasone, beclomethasone dipropionate, desonide, azathioprine, clobetasol propionate, monobenzone, fluticasone, betamethasone valerate, psoralen, and hydrocortisone valerate, and this is achieved through the use of QSPR modeling. TIs, *b*, *r*, and *N* represent the independent variable, regression model constant, correlation coefficient, and sample size, respectively. The said kind of test can be useful for comparing and deciding on model improvements. It is worth noting that *r* is higher than 0.6 and the*p*values are almost higher than 0.05. As a result, it determines that all properties are significant.

### 3.4. Standard Error of Estimate (SE), Correlation Determination, and Comparison

The standard error estimate is the measure of variation for an observation calculated around the computed regression line. It examines extent of correctness of predictions made about the calculated regression line in Table 13. In Table 14, the percentage of relationship described by correlation determination gives ample information about the relationship between variables. It is calculated by squaring the value of *r*. Tables 15–19 compare the experimental and theoretical measurement results of the models' physicochemical properties.

## 4. Conclusions

The statistical parameters used during linear QSPR models and TIs demonstrate that *ABC* (*G*) index provides high correlated value for molar volume *r* = 0.858. *F*(*G*) index offers maximum correlated value of complexity, i.e., *r* = 0.952. *M*_2_(*G*) index depicts utmost correlation coefficient of refractivity *r* = 0.971. Harmonic *H* (*G*) provides maximum correlated value of enthalpy *r* = 0.838.

The QSPR modeling is crucial because it makes physical properties more predictable. It offers a technique to do away with time-consuming experimenting and saves time. Without conducting any experiments, the elusive are anticipated. QSPR modeling is beneficial to create and forecast drug characteristics. This technique will be used to forecast in addition to create novel drugs for the future treatment of additional ailments. Getting creation of drugs is not a simple task because it may be expensive, time consuming, and difficult at times. But this approach is superior and efficient in producing the need. In this paper, we calculated TIs and compared them to a linear QSPR model for drugs used to treat vitiligo. The findings acquired in this manner would be useful in the pharmaceutical industry in inventing better drugs to obtain precautionary measures for the aforementioned disease. The correlation coefficient makes a significant contribution to the scope of TIs for such drugs. The observations are eye opening for pharmaceutical researchers working on drug science, and they offer a method to predict physicochemical properties for amateur inventions of many other specific diseases.

## Figures and Tables

**Table 1:** The TIs value drugs.

Name of drug	*ABC*	*RA*	*S*	*GA*	*M*_1_	*M*_2_	*H*	*HM*	*F*
Fluticasone propionate	22.48	13.29	13.8	29.32	162	204	12.43	886	478
Clobetasone	22.62	12.96	13.57	29.08	167	214	12.01	941	513
Beclomethasone dipropionate	28.11	16.81	17.42	36.98	204	261	15.78	1128	606
Desonide	24.48	13.98	14.85	32.2	184	238	13.11	1036	560
Clobetasol propionate	21.63	12.54	13.17	28.36	162	212	11.73	922	498
Azathioprine	14.08	8.79	9.24	19.68	96	115	8.6	474	244
Monobenzone	11.42	7.34	7.58	15.7	72	79	7.2	328	170
Betamethasone valerate	24.36	14.65	15.29	32.58	174	219	13.91	932	494
Psoralen	11.34	6.82	7.29	15.66	78	93	6.67	386	200
Hydrocortisone valerate	25.04	15.13	15.74	33.48	180	227	14.36	974	520
Fluticasone	26.65	15.83	16.43	35.03	196	254	14.81	1098	590

**Table 2:** Physical properties of drugs.

Name of drug	Refractivity (m^3^mol^−1^)	Enthalpy (C)	Molar volume (cm^3^)	Polarity (cm^3^)	Complexity	Boiling point
Fluticasone propionate	121.65	98.0	377.00	48.01	984	568.30
Clobetasone	104.72	95.3	309.10	40.50	850	549.00
Beclomethasone dipropionate	134.79	103.5	302.60	41.60	1050	600.20
Desonide	112.06	99.6	320.10	43.30	873	580.10
Clobetasol propionate	119.32	98.1	364.10	46.70	929	569.00
Azathioprine	69.94	96.9	145.40	27.30	354	685.70
Monobenzone	59.11	62.8	172.60	23.50	167	359.10
Betamethasone valerate		102.3	382.40	49.00	957	598.90
Psoralen		60.9	134.00	19.80	284	362.60
Hydrocortisone valerate	120.38	101.8	367.60	47.20	832	595.30
Fluticasone	107.87	95.9	323.20	42.40	861	553.20

**Table 3:** Statistical parameters used in QSPR model of *ABC* (*G*).

Physiochemical property	*N*	*A*	*b*	*r*	*r* ^2^	*F*	*p*	Indicator
Enthalpy	11	46.897	2.171	0.836	0.699	20.880	0.001	Significant
Polarity	11	6.498	1.426	0.621	0.386	5.658	0.041	Significant
Molar volume	11	2.954	13.765	0.858	0.736	25.032	0.001	Significant
Complexity	11	-309.258	50.191	0.945	0.893	75.089	0.000	Significant
Refractivity	9	14.804	4.199	0.920	0.846	38.376	0.000	Significant

**Table 4:** Statistical parameters used in QSPR model of *RA* (*G*).

Physiochemical property	*N*	*A*	*b*	*r*	*r* ^2^	*F*	*p*	Indicator
Enthalpy	11	-2.826	6.871	0.743	0.552	11.019	0.009	Significant
Polarity	11	5.422	2.676	0.875	0.765	29.283	0.000	Significant
Molar volume	11	-1.826	23.293	0.843	0.710	22.062	0.001	Significant
Complexity	11	-321.914	84.555	0.924	0.855	52.887	0.000	Significant
Refractivity	9	11.323	7.267	0.908	0.824	32.860	0.001	Significant

**Table 5:** Statistical parameters used in QSPR model of *S* (*G*).

Physiochemical property	*N*	*A*	*b*	*r*	*r* ^2^	*F*	*p*	Indicator
Enthalpy	11	-4.082	6.671	0.745	0.555	11.243	0.008	Significant
Polarity	11	4.913	2.599	0.877	0.770	30.112	0.000	Significant
Molar volume	11	-5.918	22.601	0.845	0.713	22.386	0.001	Significant
Complexity	11	-338.894	82.205	0.928	0.862	56.013	0.000	Significant
Refractivity	9	10.675	7.010	0.910	0.828	33.799	0.001	Significant

**Table 6:** Statistical parameters used in QSPR model of *GA* (*G*).

Physiochemical property	*N*	*A*	*b*	*r*	*r* ^2^	*F*	*p*	Indicator
Enthalpy	11	-3.676	3.112	0.747	0.559	11.388	0.008	Significant
Polarity	11	5.068	1.213	0.880	0.774	30.897	0.000	Significant
Molar volume	11	-4.316	10.537	0.846	0.716	22.713	0.001	Significant
Complexity	11	-335.797	38.423	0.932	0.870	59.991	0.000	Significant
Refractivity	9	11.940	3.243	0.912	0.832	34.670	0.001	Significant

**Table 7:** Statistical parameters used in QSPR model of *M*_1_ (*G*).

Physiochemical property	*N*	*A*	*b*	*r*	*r* ^2^	*F*	*p*	Indicator
Enthalpy	11	5.191	0.514	0.770	0.593	13.093	0.006	Significant
Polarity	11	9.228	0.196	0.886	0.784	32.691	0.000	Significant
Molar volume	11	30.594	1.708	0.856	0.732	24.595	0.001	Significant
Complexity	11	-214.531	6.269	0.949	0.900	81.168	0.000	Significant
Refractivity	9	24.456	0.513	0.920	0.846	38.420	0.000	Significant

**Table 8:** Statistical parameters used in QSPR model of *M*_2_ (*G*).

Physiochemical property	*N*	*A*	*b*	*r*	*r* ^2^	*F*	*p*	Indicator
Enthalpy	11	11.592	0.374	0.769	0.591	12.989	0.006	Significant
Polarity	11	11.709	0.142	0.883	0.779	31.732	0.000	Significant
Molar volume	11	52.287	1.240	0.853	0.727	23.987	0.001	Significant
Complexity	11	-138.898	4.569	0.950	0.902	83.061	0.000	Significant
Refractivity	9	31.763	0.368	0.971	0.841	37.087	0.000	Significant

**Table 9:** Statistical parameters used in QSPR model of *HM* (*G*).

Physiochemical property	*N*	*A*	*b*	*r*	*r* ^2^	*F*	*p*	Indicator
Enthalpy	11	13.232	0.085	0.776	0.602	13.614	0.005	Significant
Polarity	11	12.603	0.032	0.882	0.778	31.535	0.000	Significant
Molar volume	11	59.517	0.279	0.854	0.730	24.303	0.001	Significant
Complexity	11	-111.888	1.029	0.951	0.905	85.436	0.000	Significant
Refractivity	9	33.988	0.083	0.918	0.842	37.321	0.000	Significant

**Table 10:** Statistical parameters used in QSPR model of *H* (*G*).

Physiochemical property	*N*	*A*	*b*	*r*	*r* ^2^	*F*	*p*	Indicator
Enthalpy	11	43.150	4.138	0.838	0.703	21.272	0.001	Significant
Polarity	11	1.974	2.891	0.663	0.439	7.054	0.026	Significant
Molar volume	11	-11.641	25.466	0.835	0.697	20.680	0.001	Significant
Complexity	11	-355.677	92.284	0.914	0.835	45.710	0.000	Significant
Refractivity	9	7.904	7.986	0.900	0.809	29.722	0.001	Significant

**Table 11:** Statistical parameters used in QSPR model of *F* (*G*).

Physiochemical property	*N*	*A*	*b*	*r*	*r* ^2^	*F*	*p*	Indicator
Enthalpy	11	57.317	0.079	0.821	0.674	18.643	0.002	Significant
Polarity	11	14.324	0.050	0.584	0.341	4.663	0.059	Significant
Molar volume	11	65.780	0.508	0.855	0.731	24.433	0.001	Significant
Complexity	11	-88.525	1.870	0.952	0.905	86.119	0.000	Significant
Refractivity	9	35.904	0.150	0.917	0.842	37.172	0.000	Significant

**Table 12:** Correlation coefficient.

Topological index	Correlation coefficient				
	Enthalpy	Polarity	Molar volume	Complexity	Refractivity
*ABC* (*G*)	0.836	0.621	0.858	0.945	0.920
*RA* (*G*)	0.743	0.875	0.843	0.924	0.908
*S* (*G*)	0.745	0.877	0.845	0.928	0.910
*GA* (*G*)	0.747	0.880	0.846	0.932	0.912
*M*_1_ (*G*)	0.770	0.886	0.856	0.949	0.920
*M*_2_ (*G*)	0.769	0.883	0.853	0.950	0.971
*HM* (*G*)	0.776	0.882	0.854	0.951	0.918
*F* (*G*)	0.821	0.584	0.855	0.952	0.917
*H* (*G*)	0.838	0.8663	0.835	0.914	0.900

**Table 13:** Standard error of estimate.

Topological index	Std. error of the estimate				
	Enthalpy	Polarity	Molar volume	Complexity	Refractivity
*ABC* (*G*)	21.4533	5.14068	51.8856	107.768	10.49394
*RA* (*G*)	22.2725	5.34215	53.57962	125.620	11.19653
*S* (*G*)	22.1989	5.28520	53.30246	122.563	11.06695
*GA* (*G*)	22.1199	5.23301	53.02684	118.977	10.95059
*M*_1_ (*G*)	21.2492	5.11913	51.52025	104.072	10.48881
*M*_2_ (*G*)	21.2992	5.17904	51.99331	102.996	10.64628
*HM* (*G*)	21.0026	5.19160	51.74557	101.693	10.61813
*F* (*G*)	21.0026	5.19160	51.64475	101.327	10.63600
*H* (*G*)	22.7532	5.45760	54.81297	133.606	11.66503

**Table 14:** Coefficient of determination.

Topological index	Coefficient of determination				
	Enthalpy	Polarity	Molar volume	Complexity	Refractivity
*ABC* (*G*)	0.699	0.386	0.736	0.893	0.846
*RA* (*G*)	0.552	0.765	0.710	0.855	0.824
*S* (*G*)	0.555	0.770	0.713	0.862	0.828
*GA* (*G*)	0.559	0.774	0.716	0.870	0.832
*M*_1_ (*G*)	0.593	0.784	0.732	0.900	0.846
*M*_2_ (*G*)	0.591	0.779	0.727	0.902	0.841
*HM* (*G*)	0.602	0.778	0.730	0.905	0.842
*F* (*G*)	0.674	0.341	0.731	0.905	0.842
*H* (*G*)	0.703	0.439	0.697	0.835	0.809

**Table 15:** Comparison of actual and computed values.

Name of drug	Polarity of drug	Polarity computed from regression model								
		*ABC* (*G*)	*R* (*G*)	*S* (*G*)	*GA* (*G*)	*M*_1_ (*G*)	*M*_2_ (*G*)	*F* (*G*)	*H* (*G*)	*HM* (*G*)
Fluticasone propionate	48.01±0.5 cm^3^	38.55448	40.98604	40.7792	40.63316	40.98	40.677	38.224	37.90913	40.955
Clobetasone	40.50±0.5 cm^3^	38.75412	40.10296	40.18143	40.34204	41.96	42.097	39.974	36.69491	42.715
Beclomethasone dipropionate	41.60±0.5 cm^3^	46.58286	50.40556	50.18758	49.92474	49.212	48.771	44.624	47.59398	48.699
Desonide	43.30 ± 0.5 cm^3^	41.40648	42.83248	43.50815	44.1266	45.292	45.505	42.324	39.87501	45.755
Clobetasol propionate	46.70 ± 0.5 cm^3^	37.34238	38.97904	39.14183	39.46868	40.98	41.813	39.224	35.88543	42.107
Azathioprine	27.30 ± 0.5 cm^3^	26.57608	28.94404	28.92776	28.93984	28.044	28.039	26.524	26.8366	27.771
Monobenzone	35.50±0.5 cm^3^	22.78292	25.06384	24.61342	24.1121	23.34	22.927	22.824	22.7892	23.099
Betamethasone valerate	49.00±0.5 cm^3^	41.23536	44.6254	44.65171	44.58754	43.332	42.807	39.024	42.18781	42.427
Psoralen	19.80±0.5 cm^3^	22.66884	23.67232	23.85971	24.06358	24.516	24.915	24.324	21.25697	24.955
Fluticasone propionate	48.01±0.5 cm^3^	42.20504	45.90988	45.82126	45.67924	44.508	43.943	40.324	43.48876	43.771
Clobetasone	40.50±0.5 cm^3^	44.5009	47.78308	47.61457	47.55939	47.644	47.777	43.824	44.78971	47.739

**Table 16:** Comparison of actual and computed values.

Name of drug	Molar volume of drug	Molar volume from regression model								
		*ABC* (*G*)	*R* (*G*)	*S* (*G*)	*GA* (*G*)	*M*_1_ (*G*)	*M*_2_ (*G*)	*F* (*G*)	*H* (*G*)	*HM* (*G*)
Fluticasone propionate	377±5.0 cm^3^	312.3912	307.738	305.9758	304.6288	307.29	305.247	308.604	304.9014	306.711
Clobetasone	309.1±5.0 cm^3^	314.3183	300.0513	300.7776	302.1	315.83	317.647	326.384	294.2057	322.056
Beclomethasone dipropionate	302.6±5.0 cm^3^	389.8882	389.7293	387.7914	385.3423	379.026	375.927	373.628	390.2125	374.229
Desonide	320.1±5.0 cm^3^	339.9212	323.8101	329.7069	334.9754	344.866	347.407	350.26	322.2183	348.561
Clobetasol propionate	364.1±5.0 cm^3^	300.691	290.2682	291.7372	294.5133	307.29	315.167	318.764	287.0752	316.755
Azathioprine	145.4±7.0 cm^3^	196.7652	202.9195	202.9152	203.0522	194.562	194.887	189.732	207.3666	191.763
Monobenzone	172.6±3.0 cm^3^	160.1503	169.1446	165.3976	161.1149	153.57	150.247	152.14	171.7142	151.029
Betamethasone valerate	382.4±5.0 cm^3^	338.2694	339.4165	339.6513	338.9795	327.786	323.847	316.732	342.5911	319.545
Psoralen	134.0±5.0 cm^3^	159.0491	157.0323	158.8433	160.6934	163.818	167.607	167.38	158.2172	167.211
Hydrocortisone valerate	367.6±5.0 cm^3^	347.6296	350.5971	349.8217	348.4628	338.034	333.767	329.94	354.0508	331.263
Fluticasone	336.6±5.0 cm^3^	369.7913	366.9022	365.4164	364.7951	365.362	367.247	365.5	365.5105	365.859

**Table 17:** Comparison of actual and computed values.

Name of drug	Enthalpy of drug	Enthalpy from regression model								
		*ABC* (*G*)	*R* (*G*)	*S* (*G*)	*GA* (*G*)	*M*_1_ (*G*)	*M*_2_ (*G*)	*F* (*G*)	*H* (*G*)	*HM* (*G*)
Fluticasone propionate	98.0±6.0°C	88.98652	88.48959	87.9778	87.56784	88.459	87.888	53.862	94.58534	88.542
Clobetasone	95.3±6.0°C	89.54988	86.22216	86.44347	86.82096	91.029	91.628	97.844	92.84738	93.217
Beclomethasone dipropionate	103.5±6.0°C	111.6416	112.6755	112.1268	111.4058	110.047	109.206	105.191	108.4476	109.112
Desonide	99.6±6.0°C	97.03452	93.23058	94.98235	96.5304	99.767	100.604	101.557	97.39918	101.292
Clobetasol propionate	98.1±6.0°C	85.56612	83.33634	83.77507	84.58032	88.459	90.88	96.659	91.68874	91.602
Azathioprine	96.9±3.0°C	55.18492	57.57009	57.55804	57.56816	54.535	54.602	76.593	78.7368	53.522
Monobenzone	62.8±3.0°C	44.48108	47.60714	46.48418	45.1824	42.199	41.138	70.747	72.9436	41.112
Betamethasone valerate	102.3±6.0°C	96.55164	97.83415	97.91759	97.71296	94.627	93.498	96.343	100.7096	92.452
Psoralen	60.9±3.0°C	44.15916	44.03422	44.54959	45.05792	45.283	46.374	73.117	70.75046	46.042
Hydrocortisone valerate	101.8±6.0°C	99.28796	101.1322	100.9195	100.5138	97.711	96.49	98.397	102.5717	96.022
Fluticasone	95.9±6.0°C	105.7666	105.9419	105.5225	105.3374	105.935	106.588	103.927	104.4338	106.562

**Table 18:** Comparison of actual and computed values.

Name of drug	Refractivity of drug	Refractivity from regression model								
		ABC (G)	*R* (*G*)	*S* (*G*)	*GA* (*G*)	*M*_1_ (*G*)	*M*_2_ (*G*)	*F* (*G*)	*H* (*G*)	*HM* (*G*)
Fluticasone propionate	121.65 cm^3^	109.1975	107.9014	107.413	107.0248	107.562	106.835	107.604	107.17	107.526
Clobetasone	104.72 cm^3^	109.7854	105.5033	105.8007	106.2464	110.127	110.515	112.854	103.8159	112.091
Beclomethasone dipropionate	134.79cm^3^	132.8379	133.4813	132.7892	131.8661	129.108	127.811	126.804	133.9231	127.612
Desonide	112.06 cm^3^	117.5955	112.9157	114.7735	116.3646	118.848	119.347	119.904	112.6005	119.976
Clobetasol propionate	119.32cm^3^	105.6284	102.4512	102.9967	103.9115	107.562	109.779	110.604	101.5798	110.514
Azathioprine	59.94 cm^3^	73.92592	75.19993	75.4474	75.76224	73.704	74.083	72.504	76.5836	73.33
Monobenzone	59.11 cm^3^	62.75658	64.66278	63.8108	62.8551	61.392	60.835	61.404	65.4032	61.212
Betamethasone valerate		117.0916	117.7846	117.8579	117.5969	113.718	112.355	110.004	118.9893	111.344
Psoralen		62.42066	60.88394	61.7779	62.72538	64.47	65.987	65.904	61.17062	66.026
Hydrocortisone valerate	120.38cm^3^	119.947	121.2727	121.0124	120.5156	116.796	115.299	113.904	122.583	114.83
Fluticasone	107.87cm^3^	126.7074	126.3596	125.8493	125.5423	125.004	125.235	124.404	126.1767	125.122

**Table 19:** Comparison of actual and computed values.

Name of drug	Complexity of drug	Complexity from regression model								
		ABC(G)	R (G)	S(G)	Ga(G)	M1(G)	M2(G)	F(G)	H(G)	HM(G)
Fluticasone propionate	984	819.0357	801.822	795.535	790.7654	801.047	793.178	805.335	791.4131	799.806
Clobetasone	850	826.0624	773.9188	776.6279	781.5438	832.392	838.868	870.785	752.6538	856.401
Beclomethasone dipropionate	1050	1101.611	1099.456	1093.117	1085.086	1064.345	1053.611	1044.695	1100.565	1048.824
Desonide	873	919.4177	860.1649	881.8503	901.4236	938.965	948.524	958.675	854.1662	954.156
Clobetasol propionate	929	776.3733	738.4057	743.7459	753.8793	801.047	829.73	842.735	726.8143	836.85
Azathioprine	354	397.4313	421.3245	420.6802	420.3676	387.293	386.537	367.755	437.9654	375.858
Monobenzone	167	263.9232	298.7197	284.2199	267.4441	236.837	222.053	229.375	308.7678	225.624
Betamethasone valerate	957	913.3948	916.8168	918.0205	916.0243	876.275	861.713	835.255	927.9934	847.14
Psoralen	284	259.9079	254.7511	260.3805	265.9072	274.451	286.019	285.475	259.8573	285.306
Hydrocortisone valerate	832	947.5246	957.4032	955.0127	950.605	913.889	898.265	883.875	969.5212	890.358
Fluticasone	861	1028.332	1016.592	1011.734	1010.161	1014.193	1021.628	1014.775	1011.049	1017.954

## Data Availability

The data used to support the findings of this study are included within the article.
